# Delayed Diagnosis of Congenital Adrenal Hyperplasia Due to 3β-Hydroxysteroid Dehydrogenase Type 2 Deficiency

**DOI:** 10.1210/jcemcr/luaf212

**Published:** 2025-10-09

**Authors:** Saira Yousaf, Ryizan Nizar, George Alias, David Taylor, Piers Fulton, Thalia Antoniadi

**Affiliations:** Department of Diabetes and Endocrinology, Great Western Hospital NHS Foundation Trust, Swindon SN3 6BB, UK; Department of Diabetes and Endocrinology, Great Western Hospital NHS Foundation Trust, Swindon SN3 6BB, UK; Department of Endocrinology, Sheikh Khalifa Hospital, Fujairah, UAE; Department of Diabetes and Endocrinology, Great Western Hospital NHS Foundation Trust, Swindon SN3 6BB, UK; Department of Clinical Biochemistry (Synnovis), Kings College Hospital NHS Foundation Trust, London SE5 9RS, UK; West Midlands Regional Genomics Laboratory, Birmingham Women's and Children's NHS Foundation Trust, Brimingham B15 2TG, UK; West Midlands Regional Genomics Laboratory, Birmingham Women's and Children's NHS Foundation Trust, Brimingham B15 2TG, UK

**Keywords:** congenital adrenal hyperplasia, CAH, 3β-hydroxysteroid dehydrogenase, 3β-HSD2 deficiency, salt wasting, SW

## Abstract

Congenital adrenal hyperplasia (CAH) is an autosomal recessive condition associated with enzymatic deficiencies affecting the adrenal steroid synthesis pathway. 21-hydroxylase deficiency (21-OHD), the most common form, is a pathogenic variant of the *CYP21A2* gene and accounts for >95% of cases. Deficiency of 3β-hydroxysteroid dehydrogenase type 2 (3β-HSD2) is a rare form, accounting for <0.5% of cases. Detection of 3β-HSD deficiency depends on the methods used by laboratories for screening of CAH. We present the case of a 46-year-old female who was diagnosed with 21-OHD CAH at birth. As an adult, she was reviewed in a routine endocrine clinic and showed signs of hyperandrogenism, including hirsutism and male pattern hair loss. She was on prednisolone 3 mg/day and fludrocortisone 200 mcg/day. Trials of multiple antiandrogenic therapies did not help with hirsutism. 17-hydroxyprogesterone concentration was unexpectedly low, despite the low-dose glucocorticoid treatment. This raised our suspicion of an alternative etiology. Further investigations were undertaken, including a 24-hour urinary steroid profile and genetic testing, which led to the diagnosis of 3β-HSD2 deficiency. This case highlights that clinical suspicion of rarer forms of CAH is necessary to identify these cases, as they can lead to delayed diagnosis and treatment and inadequate glucocorticoid replacement.

## Introduction

Congenital adrenal hyperplasia (CAH) is an autosomal recessive condition that is associated with enzymatic deficiencies affecting different steps of the steroid synthetic pathway. Mutation of the *CYP21A2* gene, which encodes the enzyme 21-hydroxylase (21-OH), is the most common cause of CAH, accounting for >95% of cases, followed by a pathogenic variant of 11β-hydroxylase (*CYP11B1*) and, more rarely, other enzymes, including 3β-hydroxysteroid dehydrogenase type 2 (3β-HSD2). All forms of CAH lead to impaired glucocorticoid (cortisol) production and, depending on the CAH form and severity, accompanying mineralocorticoid deficiency.

Clinical presentation of CAH can vary significantly depending on the severity of the enzyme deficiency. 21-OHD CAH is typically categorized into 3 phenotypes: salt-wasting (SW) CAH, simple-virilizing CAH, and nonclassic CAH. The SW type presents in the neonatal period with SW crisis. Female infants typically develop varying degrees of virilization, including atypical genitalia. Male infants may have normal phenotypical features at birth but may also have subtle findings like enlarged phallus or hyperpigmentation around the scrotum. If not identified during neonatal screening, they present with failure to thrive.

Simple-virilizing CAH leads to marked androgen excess with virilization and contrasexual precocity in females and GnRH-independent precocity in males. In contrast, nonclassic CAH is typically associated with a milder degree of hyperandrogenism and may present with hirsutism, acne, menstrual irregularities, subfertility, or infertility.

The signs and symptoms of androgen excess in CAH are the result of an accumulation of adrenal androgens, including dehydroepiandrosterone (DHEA) and androstenedione. These are formed secondary to the shunting of increased levels of steroid synthetic pathway intermediates into the adrenal androgen pathway, with the most notable of these in both 21-OH and 11β-hydroxylase deficiencies being 17-hydroxyprogesterone (17-OHP). Several countries have implemented screening programs for 21-OHD in neonates, using 17-OHP as the key indicator of the disease [1].

**Figure 1.**
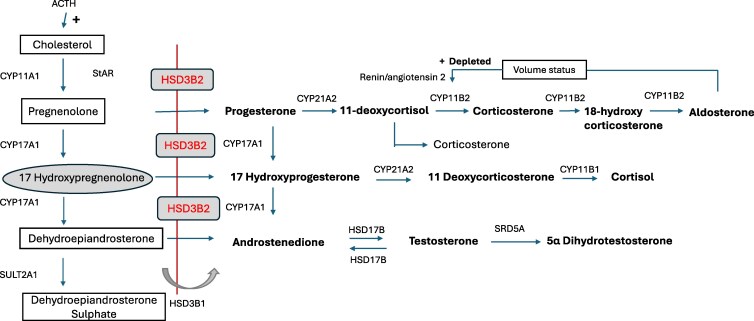
Steroidogenesis in adrenal cortex. Pathophysiology in 3β–hydroxysteroid dehydrogenase type 2 deficiency [2]. Image adapted and modified from Endocrine. 2019 Feb 4;63(3):407-421. doi: 10.1007/s12020-018-01835-3 (https://creativecommons.org/licenses/by/4.0/).

A rarer form of CAH is due to 3β-HSD2 deficiency, accounting for <0.5% of all CAH cases [2]. 3β-HSD has 2 isozymes—3β-HSD type 1 (HSD3B1) and type 2 (HSD3B2)—which share 93.5% sequence identity. Both genes (*HSD3B1* and *HSD3B2*) are located on chromosome 1p13.1 [3]. HSD3B2 is expressed in the adrenal glands and in gonadal tissue, where it catalyzes the conversion of pregnenolone, 17-hydroxypregnenolone, and DHEA to progesterone, 17-OHP, and androstenedione, respectively [4]. In contrast, HSD3B1 is primarily expressed in the placenta, skin, and peripheral tissues and converts DHEA to androstenedione (Fig. 1) [2, 5]. The ability of newborn screening to detect 3β-HSD2 CAH cases depends largely on the methodology used by laboratories for CAH detection. If a 17-OHP immunoassay is used, the 17-hydroxypregenenolone that accumulates in 3β-HSD2 deficiency CAH may cross-react and give a positive screening result for 21-OHD. However, if a more specific mass spectrometry method is subsequently used for confirmation, this may give a negative screen, despite some 17-hydroxypregnenolone being converted peripherally to 17-OHP by HSD3B1.

We report a case of delayed diagnosis of 3β-HSD2 deficiency in a 46-year-old female, diagnosed at birth with CAH secondary to 21-OHD.

## Case Presentation

A 46-year-old woman was referred to the endocrine clinic for follow-up of her CAH. She had been diagnosed with CAH due to 21-OH deficiency at birth following signs and symptoms of SW. We did not have access to her medical records before 2003; therefore, details of the basis on which the original diagnosis had been made were not known. As per her father, there was no reported history of atypical genitalia or differences in sex development at birth.

Her past medical history included lipoid proteinosis, post-mumps meningitis, epilepsy, and cardiac arrhythmias leading to the fitting of a pacemaker. In terms of her family history, she had no siblings or children of her own. Her parents were first cousins.

On clinical examination at follow-up, she showed signs of hyperandrogenism, including hirsutism and male pattern hair loss. To address her symptoms, she had been prescribed medications including spironolactone, cyproterone acetate, and finasteride, which, she reported, had been of little clinical benefit. She was also established on prednisolone 1 mg in the morning and 2 mg in the evening, fludrocortisone 200 mcg once a day, oestradiol/dydrogesterone 1/10 mg once a day, and levetiracetam 1 gm twice a day.

## Diagnostic Assessment

As part of regular monitoring, she had her hormonal profile checked, as detailed in Table 1. What was striking was how low the 17-OHP concentration was for a patient with 21-OHD CAH on such a small dose of prednisolone and with a high ACTH. This raised suspicion for an alternate etiology of CAH. To look into this further, we organized a 24-hour urinary steroid profile and genetic testing.

**Table 1.** Hormonal profile

Test	Plasma levels	Reference range
17-OH progesterone	0.7 nmol/L (23.1322 ng/dL)	Follicular stage 0.4-3.6 nmol/L Luteal stage 1.2-6.0 nmol/L
Androstenedione	1.7 nmol/L (48.68 ng/dL)	0.9-4.8 nmol/L (25.776-137.472 ng/dL)
Testosterone	0.8 nmol/L (23.07 ng/dL)	0.3-3.1 nmol/L (8.6-89.4 ng/dL)
Sodium	138 mmol/L (138 mEq/L)	133-146 mmol/L (133-146 mEq/L)
HbA1c	43 mmol/mol (7.1%)	<40 mmol/mol (<5.7%)
TSH	2.82 mIU/L (2.82 µIU/mL)	0.35-5.50 mIU/L (0.35-5.50 µIU/mL)
ACTH	1365 ng/L (300 pmol/L)	0-46 ng/L (0-10.1 pmol/L)
DHEA	5.59 µmol/L (2.0597 mg/L)	0-5 µmol/L (0- 1.8423 mg/L)
Renin	16.5 mIU/L (9.9 pg/mL)	5.4-30 mIU/L (3.24-18 pg/mL)
Chromosomal analysis	46 XX Normal female karyotype	

Abbreviations: 17-OH progesterone, 7-hydroxyprogesterone; DHEA, dehydroepiandrosterone; HbA1c, glycated hemoglobin.

**Figure 2.**
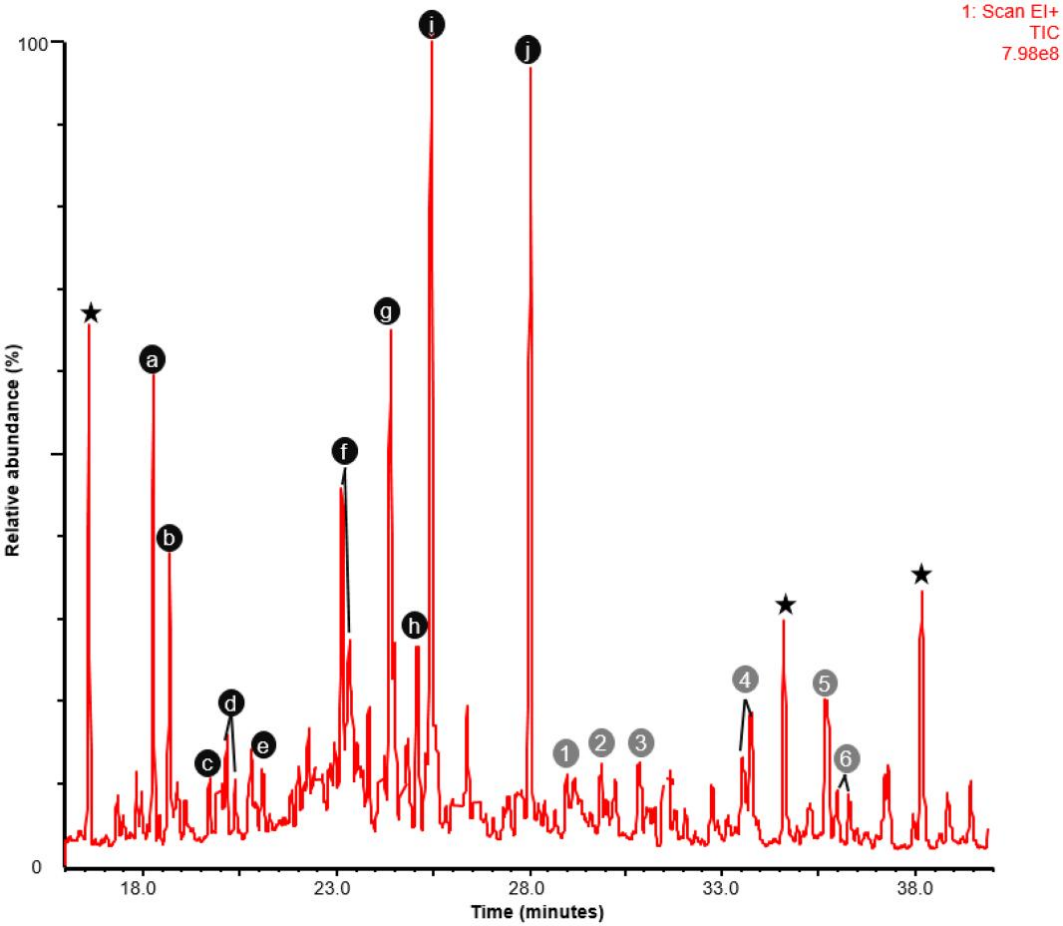
Urine steroid profile total ion current chromatogram from gas chromatography-mass spectrometry analysis. Endogenous steroid metabolites labeled by black lettered circles are derived from androstenedione and testosterone [androsterone (A) and aetiocholanolone (B)], DHEA [(DHEA (C), 3α,16α-OH-DHA (D), 16α-OH-DHA (F), 16-oxoandrostenediol (H), and androstenetriol (I)], 17-hydroxyprogesterone [17-hydroxypregnanolone (E) and pregnanetriol (G)] and 17-hydroxypregnenolone [pregnenetriol (J)]. Prednisolone metabolites detected are labeled by grey numbered circles [1-6]. The black stars indicate the position of exogenous internal standards. Abbreviations: 3α,16α-OH-DHA, 3α,16α-hydroxydehydroepiandrosterone, 16α-OH-DHA, 16α-hydroxydehydroepiandrosterone; DHEA, dehydroepiandrosterone.

Urine steroid profiling was performed while the patient was maintained on prednisolone (Fig. 2). Low levels of free and A-ring reduced prednisolone metabolites were detected (Fig. 2, labeled 2-6), while endogenous cortisol and cortisone metabolites were scarcely detected. On this background, high concentrations of metabolites of DHEA (3α,16α-hydroxydehydroepiandrosterone, 16α-hydroxydehydroepiandrosterone, 16-oxoandrostenediol, and androstenetriol; Fig. 2, labeled c, d, f, h, and I, respectively) and 17-hydroxypregnenolone (pregnenetriol; Fig. 2, labeled j) were detected. The presence of these metabolites indicated an underlying CAH due to 3β-hydroxysteroid dehydrogenase type 2 deficiency. Paradoxically for this disorder, there was also a relative increase of 17-OHP metabolites (pregnanetriol and 17-hydroxypregnanolone; Fig. 2, labeled e and g) and androstenedione/testosterone metabolites (androsterone and aetiocholanolone; Fig. 2, labeled a and b). This is a frequent urine steroid profile finding in 3β-HSD2 deficiency CAH patients on glucocorticoid replacement and is believed to reflect a relatively enhanced effect of peripheral 3β-HSD1 enzyme activity on a diminished amount of precursor. CAH due to 21-OHD is excluded by the absence in the profile of 11-oxopregnanetriol, the adrenal-specific 17-hydroxyprogesterone metabolite (derived from 21-deoxycortisol, which is formed by the activity of 11β-hydroxylase on adrenal 17-hydroxyprogesterone).

Genetic analysis of the *CYP21A2* gene was carried out to verify an initial working diagnosis. Multiplex ligation-dependent probe amplification and sequencing analysis did not detect any variants in *CYP21A2*, other than a common polymorphism of no known clinical significance. We then requested further genetic testing to include other genes associated with disorders of sex development.

Illumina next-generation sequencing analysis of a panel of genes associated with disorders/differences of sex development was captured using the Nonacus Cell3Target ExomeCG enrichment system.

The nonsense variant HSD3B2 (NM_000198.3):c.15C > A p.(Cys5*) was detected in the homozygous state. This previously reported [7] variant was classified as likely pathogenic. American College of Medical Genetics and Genomics/Association for Clinical Genomic Science variant classification criteria PVS1 (strong), PM2 (moderate), and PS4 (supporting) apply [8]. Sanger sequencing was used to confirm the presence of this variant in the affected patient and to confirm that both of her parents are heterozygous carriers.

While the genetic tests classified the variant as “likely”, the urine steroid profile was supportive of confirming the diagnosis of CAH secondary to 3β-HSD2 deficiency. Further genetic testing of the parents confirmed that they were carriers of the same variant.

## Treatment

Management of patients with 3β-HSD2 deficiency CAH follows the same principles that are used in all other types of SW CAH. Patient remained stable on current steroid and mineralocorticoid replacement with no signs and symptoms suggestive of adrenal insufficiency.

## Outcome and Follow-up

The patient was counseled regarding the diagnosis, and her family was offered genetic screening for this pathogenic variant of CAH. The patient remains under endocrine clinic follow-up.

## Discussion

We describe an unusually delayed diagnosis of a case of classical 3β-HSD2 deficiency CAH due to homozygosity for a very rare nonsense variant in the *HSD3B2* gene. We could find only 1 case report of this pathogenic variant in a female who presented with coexisting autoimmune adrenalitis [7].

The case had initially presented as SW CAH in the neonatal period and had been clinically diagnosed as 21-OHD at that time. In contrast to 21-OHD CAH, in which 17-OHP levels are principally used to aid diagnosis, the diagnosis of 3β-HSD2 deficiency can be much more challenging.

This case highlights the usefulness of urine steroid profiling in confirming the CAH subtype diagnosis, as in this case a genetic test was not conclusive but indicated “likely” pathogenic.

Management of patients with 3β-HSD2 deficiency CAH follows the same principles that are used in all other forms of SW CAH. The main aim of treatment is to achieve adequate physiological glucocorticoid and mineralocorticoid replacement. In children, hydrocortisone replacement doses of 10 to 15 mg/m^2^/day are used, and in adults longer-acting steroid doses can also be used (eg, dexamethasone or prednisolone), as they are known to suppress growth in children [2]. For mineralocorticoid replacement, fludrocortisone 0.1 mg/day can be used with regular monitoring of plasma renin activity to guide adequate replacement [2]. Careful monitoring is required to avoid iatrogenic hypercortisolism and its associated comorbidities. It is also important to reduce exposure to adrenal androgens according to age and sex.

In contrast to 21-OHD CAH, where 17-OHP measurement can reliably be used for both diagnosis and treatment monitoring, biochemical diagnosis and monitoring for 3β-HSD2 deficiency CAH are challenging. In the neonatal period, patients can be incorrectly diagnosed with 21-OHD CAH, as was the case in our patient [1]. Studies suggest that in a proportion of patients, 17-OHP may be elevated in neonates with 3β-HSD2 deficiency, which may be due to conversion of 17-hydroxypregenolone to 17-OHP by 3β-HSD1 in peripheral tissues and placenta. In addition, 17-hydroxpregnenolone-sulphate itself is a known interferent in 17-OHP immunoassays that are often employed in newborn screening programs. On the other hand, patients with known 3β-HSD2 deficiency CAH usually have low or normal levels of 17-OHP, testosterone, androstenedione, especially if measured by mass spectrometry rather than immunoassay [9]. In contrast to 21-OHD CAH, there is no readily available analogous test to 17-OHP in blood for 3β-HSD2 CAH. The most suitable steroid for diagnosis and monitoring would be 17-hydroxypregnenolone, but it is not readily available in clinical laboratories in many countries, owing to the small demand due to the rarity of the disorder. Measuring levels of DHEA/DHEAs in serum may be a more widely available alternative to consider, but there is little if any evidence of its utility in 3β-HSD2 deficiency. A regular urinary steroid profile can also be considered for monitoring; however, there is no evidence base for this. This makes the management challenging and poses a risk of over- or underreplacement of glucocorticoids and mineralocorticoids, which leads to risks of complications including obesity, diabetes, hypertension, reduced adult height, infertility, osteoporosis, and testicular adrenal rest tumors [10].

This case highlights the fact that clinical suspicion of rarer forms of CAH is necessary to identify these cases as they can lead to delayed diagnosis and treatment and inadequate glucocorticoid replacement. An incorrect diagnosis can also lead to serious implications for family members if appropriate screening is not offered. To the best of our knowledge, there is only 1 case report in the literature that describes this genetic variant of 3β-HSD2 deficiency.

## Learning Points

- Patients with known 3β-HSD2 deficiency CAH usually have low or normal levels of 17-OHP, testosterone, and androstenedione, especially if measured by mass spectrometry rather than immunoassay.
- 17-hydroxpregnenolone-sulphate itself is a known interferent in 17-OHP immunoassays that are often employed in newborn screening programs.
- Clinical suspicion of rarer forms of CAH is necessary to identify rarer forms of CAH as they can lead to delayed diagnosis and treatment and inadequate glucocorticoid replacement.
- Incorrect diagnosis can also lead to serious implications for family members if appropriate screening is not offered.

## Contributors

All authors made individual contributions to authorship. R.N.: diagnosis and management of patient and preparation of initial draft. G.A.: data collection, review of draft, and patient follow-up. S.Y.: literature review, report writing, and manuscript submission. D.T.: urine steroid profiles analysis. P.F., T.A.: genetic analysis. All authors reviewed and approved the final draft.

## Data Availability

Data sharing is not applicable to this article as no datasets were generated or analyzed during the current study.
